# Predisposition of Hip Prosthesis Component Positioning on Dislocation Risk: Biomechanical Considerations Based on Finite Element Method Analysis

**DOI:** 10.3390/jcm14197056

**Published:** 2025-10-06

**Authors:** Maciej Kostewicz, Marcin Zaczyk, Grzegorz Szczęsny

**Affiliations:** 1 Department of Orthopedics and Traumatology of the Musculoskeletal System, Medical University of Warsaw, 02-091 Warsaw, Poland; grzegorz.szczesny@wum.edu.pl; 2 Institute of Micromechanics and Photonics, Faculty of Mechatronics, Warsaw University of Technology, 02-525 Warsaw, Poland; macin.zaczyk@pw.edu.pl

**Keywords:** total hip arthroplasty, dislocation, finite element method, numerical analysis

## Abstract

**Background/Objectives:** Total hip arthroplasty (THA) is a widely accepted and effective intervention for advanced degenerative hip disease. However, prosthetic dislocation remains one of the most common postoperative complications. This study aimed to evaluate the biomechanical consequences of implant positioning variations and their influence on prosthetic stability. **Methods:** A three-dimensional finite element model (FEM) of the pelvis and hip joint was developed using SolidWorks Professional 2025, based on CT imaging of an anatomically normal adult. Multiple implant configurations were simulated, varying acetabular cup inclination and anteversion angles, femoral stem depth, and femoral offset. Muscle force vectors replicating single-leg stance conditions were applied according to biomechanical reference data. The mechanical performance of each configuration was quantified using the safety factor (SF), defined as the ratio of allowable material stress to calculated stress in the model. **Results:** The configuration with 45° cup inclination, 15° anteversion, standard femoral offset, and optimal stem depth demonstrated the highest SF values (9–12), indicating a low risk of mechanical failure or dislocation. In contrast, malpositioned implants—particularly those with low or high anteversion, excessive offset, or shallow stem insertion—resulted in a marked decrease in SF values (2–5), especially in the anterosuperior and posterosuperior quadrants of the acetabular interface. **Conclusions:** The findings underscore the critical importance of precise implant alignment in THA. Even moderate deviations from optimal positioning can substantially compromise biomechanical stability and increase the risk of dislocation. These results support the need for individualized preoperative planning and the use of assistive technologies during surgery to enhance implant placement accuracy and improve clinical outcomes.

## 1. Introduction

Total hip arthroplasty (THA) is the most widely used surgical procedure nowadays, restoring joint function by replacing anatomical components that were damaged by pathological processes in advanced osteoarthritis. It enables almost immediate release of pain relief and recovery of limb function impaired by the disease. Due to its high efficacy the procedure enjoys widespread global popularity. It is estimated that approximately one million THA procedures are performed annually [1].

Although the procedure is relatively safe, it is not without the risk of complications. Dislocation is considered one of the more commonly reported mechanical complications following total hip arthroplasty, with incidence rates ranging from 0.2% to 10% depending on surgical approach, implant selection, and patient-related factors [2]. In many cases, dislocation necessitates additional intervention, most often closed reduction, and in some cases revision surgery. Dislocations most commonly occur within the first 6–12 weeks after prosthesis implantation and are frequently the result of improper positioning of components or excessive impingement between the femoral head and acetabular cup [3].

**Figure 1:**
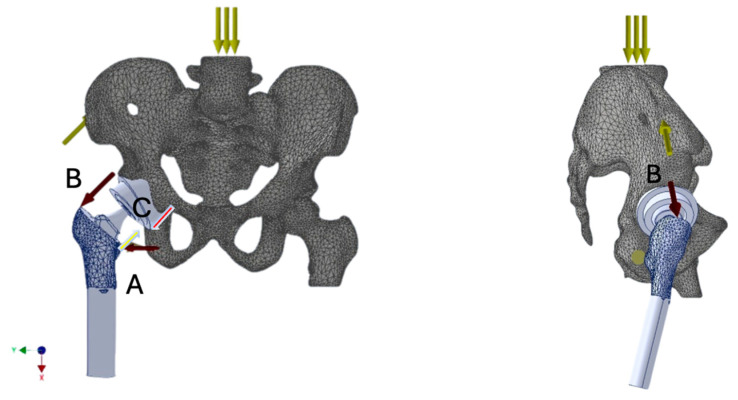
Muscle force vectors analyzed in the study acting on the prosthetic hip joint: adductor group (**A**), gluteal muscles (**B**), and iliopsoas muscle (**C**). Load distribution during single-leg stance is presented (↓↓↓).

Understanding the biomechanics of the prosthetic hip joint is crucial for establishing optimal implantation principles. Such knowledge enables analysis of the distribution of forces acting on the prosthesis during limb loading. The direction and magnitude of these forces can compromise joint stability and, under certain external conditions, predispose to dislocation. These forces result from both the mechanical load transmitted through the joint and the tension generated by the activity of specific muscle groups, primarily the quadriceps femoris, iliopsoas, gluteus minimus and medius and indirectly maximus as well, adductors, and rotators [Figure 1]. Repetitive sub-failure cycles can accumulate micro-damage and reshape local stress–strain fields, thereby elevating fatigue failure risk. Recent data-driven work demonstrates that spatial–temporal patterns extracted from these fields can predict ligament fatigue mechanisms and failure risk, complementing conventional finite-element analyses [4].

The amplitude and timing of their contractions are precisely coordinated by the nervous system, producing a resultant force that enables the execution of desired movements, stable support, and effective weight transfer [5].

Dislocations of hip prostheses are among the most frequent complications of total hip arthroplasty. They result in the rupture of soft tissues stabilizing the prosthetic joint, including the joint capsule and the scar tissue forming at the site of iatrogenic injury during implantation, as well as muscular structures and their attachments. Consequently, neurovascular structures may also be secondarily affected. These complications often lead to joint instabilities and recurrent dislocations.

Decades of clinical observations and analyses have facilitated the development of what is currently considered the optimal method for prosthesis implantation. The key principle is the accurate restoration of the anatomical parameters of the native hip. These parameters include limb length, acetabular inclination angle (the angle of the acetabular cup in the coronal plane relative to the horizontal plane), acetabular anteversion, femoral stem antetorsion, and the offset (the distance from the center of the femoral head to the femoral shaft axis). It has been observed that adherence to these principles minimizes dislocation risk, whereas deviations increase the risk of dislocation. This is attributed to the preservation (or lack thereof) of the equilibrium of mechanical forces acting on the implant. An imbalance in force vectors and amplitudes can lead to instability and, ultimately, dislocation due to loss of contact between the prosthetic components.

It should be noted that the acceptable range for each implantation parameter is relatively broad (e.g., acetabular inclination of 45° ± 10°). This variability reflects the significant role of external forces—such as trauma from stumbling, falling down, or strong impacts—in the pathogenesis of dislocation. Avoidance of such events allows for greater tolerance in component positioning while still maintaining joint stability.

The aim of this study was to demonstrate the differences in mechanical loads within the prosthetic hip joint implanted according to standard anatomical principles (acetabular inclination 45°, anteversion 15°; standard offset; and femoral stem depth placing the prosthesis rotation center at the level of the contralateral hip joint) compared to a prosthesis implanted with deviations from these principles: 60° acetabular inclination, anteversion of 0° and 30°, increased offset by 1 cm, and insufficient femoral stem depth by 2 cm. The analysis was conducted using numerical boundary problem-solving techniques and 3D interpolation within the finite element method framework.

## 2. Materials and Methods

A three-dimensional model of the pelvis and hip joints was obtained by processing sample pelvic computer tomography (CT) images of an individual without anatomical abnormalities. The DICOM data were imported and processed using Scilab 2024.0 (Esi Group), where filtering and segmentation procedures were applied to isolate bone tissue. This enabled the creation of a detailed 3D reconstruction of the bony pelvis.

CT data were processed in Scilab 2024.0, and the grayscale values (Hounsfield units) were converted into bone density values using phantom-based calibration (QRM-BDC, QRM GmbH, Möhrendorf, Germany), ensuring accurate mapping of local mechanical properties via Keller’s equation.

Material properties were then assigned to individual bone structures using Keller’s equation, which accurately reflects mechanical characteristics based on the following formula [6]:

*E* = *a*·*ρ^b^*;

where

*E*—Young’s modulus [MPa].

*a*, *b*—empirically determined coefficients, depending on the type of tissue, defined separately for trabecular and cortical bone. In this study, values of *a* = 10.5 and *b* = 2.57 were adopted.

*ρ*—density of the modeled bone tissue [g/cm^3^]. The adopted value in this study was ρ = 1.2 g/cm^3^.

*E* = 10.5·(1.2)^2.57 GPa ≈ 17.4 GPa

This value was found to be consistent with those reported in the literature [7].

By utilizing grayscale values from computer tomography (CT) images, a distribution of material groups was achieved within the 3D model. This allowed the assignment of local material properties to individual finite elements based on their corresponding local tissue density. Consequently, it was possible to compute a local, equivalent Young’s modulus for various bone regions according to their actual densities. Subsequently, an appropriate boundary conditions were then defined, constraining the translational and rotational degrees of freedom at anatomically defined fixed points, the sacroiliac joints and the pubic symphysis, reflecting pelvic stability during single-leg stance. External loads, including body weight and muscle force vectors acting on the hip joint, were considered to replicate physiological conditions as closely as possible.

A simplified contact modeling approach was applied. As a result, a biomechanical mathematical model was constructed to simulate phenomena occurring at the interface between the implant and the surrounding bone structures.

Based on numerical modeling and simulations conducted using the finite element method (FEM), the actual geometry of the hip joint was reconstructed. The developed model, created in SolidWorks Professional 2025 (Dassault Systèmes SolidWorks Corp., Waltham, MA, USA), served as the basis for strength analysis. Prosthetic components were virtually implanted using 3D modeling tools, simulating a total hip arthroplasty procedure and resulting in a hip joint model with the endoprosthesis in situ.

**Figure 2:**
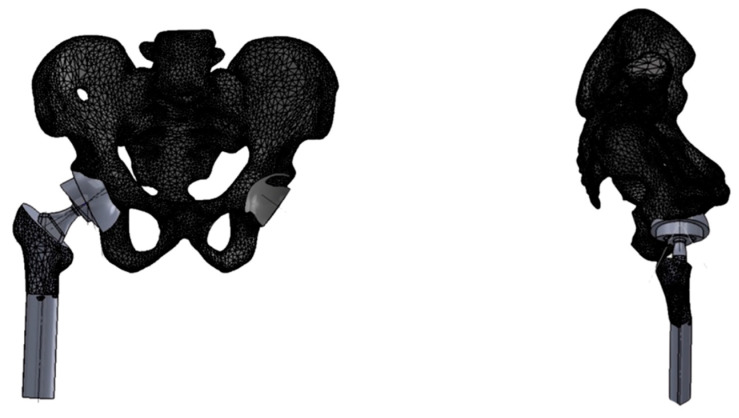
Three-dimensional model of pelvis with implanted endoprosthesis used in current work. Antero-posterior (**left**) and lateral (**right**) views.

For the purpose of analysis, parameters of a standard uncemented femoral stem (Medgal Hip, Medgal, Poland) were used, with a 52 mm hemispherical acetabular cup and a 32 mm spherical femoral head. The initial positioning of the acetabular cup was set at 45° inclination and 15° anteversion [Figure 2].

The prosthesis components were modeled as linearly elastic and isotropic. Material properties corresponding to Ti-6Al-4V were considered for both the titanium alloy stem and cup, i.e., Young’s modulus E = 110 GPa. For the CoCrMo alloy head, E = 210 GPa was assumed, and for the UHMWPE polyethylene insert, E = 1.0 GPa.

A series of implantation scenarios were developed for the prosthesis within the hip joint, varying the positioning of both the acetabular cup in the pelvis and the femoral stem within the proximal femur. These scenarios were prepared as mathematical models for finite element simulations. The computational solvers used were FFEPlus and Direct Sparse provided by the SolidWorks engineering suite.

Simplified reference conditions were applied in the model consisting of a global gravitation force and local muscle force vectors corresponding to specific muscle groups. The bone structures were modeled using a homogeneous orthotropic material, while the prosthetic components were assigned material properties based on their real-life compositions.

Muscle force models acting on bone structures (with prosthetic elements implanted) were developed as resultant force vectors representing the load on the hip joint during single-leg stance. The muscle groups and their roles included:

- Gluteal muscles—thigh abduction and primary stabilizers of the pelvis in the coronal plane;
- Iliopsoas—hip flexor;
- Adductor muscles—thigh adduction and internal rotation.

The muscle force vector values were adopted from literature sources [8], using an average force equivalent to 3–4 times body weight, expressed in Newtons (N).

The study was designed as a comparative analysis of multiple implantation scenarios (varying cup positioning, stem insertion depth, and femoral head offset) under consistent reference conditions—identical body weight loads, muscle force magnitudes, and muscle attachment locations. Each simulation aimed to visualize the mechanical load distribution—namely stress, strain, and resulting deformations—as well as the safety factor (SF) within the analyzed anatomical region (pelvis, hip joint, and proximal femur).

The objective was to assess whether these values could exceed critical thresholds, potentially leading to prosthetic component dislocation. The analysis specifically focused on the orientation of the acetabular component (inclination, anteversion) and the femoral stem (depth of insertion, offset) in the proximal femur.

The primary parameter evaluated was the Safety Factor (SF), which quantifies the structural strength margin as the ratio between the maximum allowable load and the anticipated operational load on the prosthetic components. The selection of SF as a core evaluation metric reflects its universality and its ability to clearly delineate the boundary between the safe usage and the risk of structural failure. It is also a direct indicator of biomechanical durability and potential implant dislocation risk.

The Safety Factor was not used as a direct predictor of dislocation, but rather as a biomechanical indicator of localized overload or instability at the implant-bone and implant-liner interfaces. While dislocation is a multifactorial clinical event involving soft tissue, neuromuscular control, and component alignment, it is biomechanically linked to localized stress concentrations that can result in loss of contact between articulating surfaces (e.g., femoral head and polyethylene liner). In our FEM simulations, regions with low SF values (<3) consistently correlated with mechanical configurations known from clinical literature to be associated with a higher dislocation risk (e.g., excessive anteversion, shallow stem insertion, increased offset).

**Figure 3:**
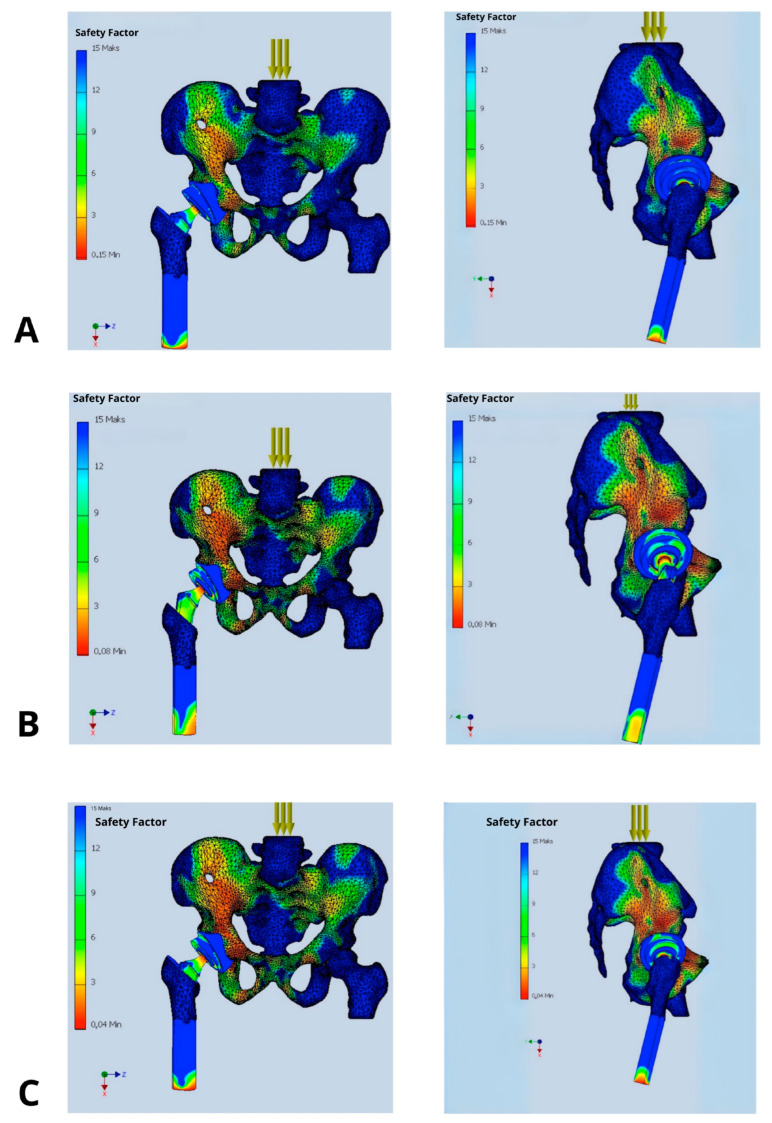

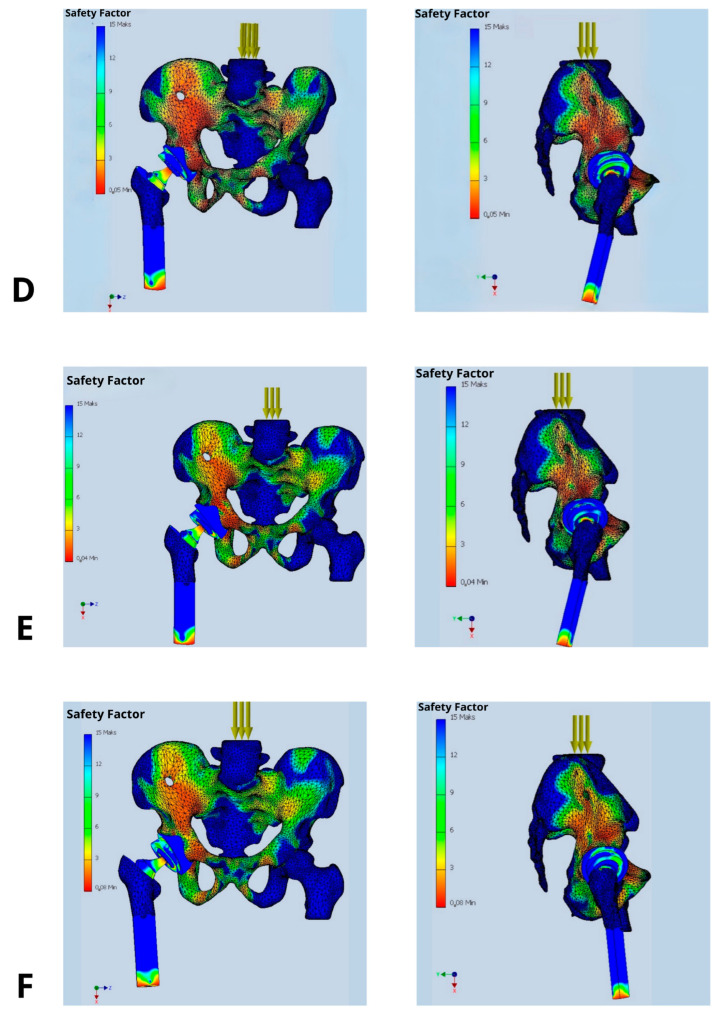
Distribution of Safety Factor within the hip prosthesis in different implant’s configurations: (**A**) implantation recommended by the manufacturer (acetabular inclination 45°, anteversion 15°, standard offset, and femoral stem depth positioning the prosthetic axis at the level of the contralateral hip joint), (**B**) femoral stem inserted 2 cm too shallowly, (**C**) acetabular inclination increased above 50°, (**D**) acetabular anteversion increased to 30°, (**E**) acetabular anteversion reduced to 0°, and (**F**) femoral head offset increased by 1 cm.

In the presented model, SF allowed a clear interpretation of how changes in prosthetic component orientation (cup and stem) affect mechanical loading. It also identifies implantation variants that fail to provide adequate safety margins [Figure 3]. Clinically, SF is a key factor while choosing optimal implant placement strategies and may also direct the proper decisions regarding surgical technique or implant design modifications. SF values in the range of 2.0–3.0 are considered associated with a high risk of mechanical failure, whereas values exceeding 6.0 are deemed safe and favorable for long-term implant integrity [9].

Particular attention was paid to the contact zone between the femoral head and the polyethylene liner of the prosthesis—i.e., the critical area where dislocation typically occurs.

## 3. Results

In the model with proper position of implant components the Safety Factor (SF) ranged from 9 to 12. Deviations from this optimal configuration resulted in a decrease in SF values:

1. In the model with femoral stem implanted 2 cm too shallowly, SF values dropped to 2–3 in the anterosuperior and anteroinferior quadrants of the acetabulum.
2. With acetabular inclination increased beyond 50°, SF values decreased to 3–4 in the anterosuperior and posterosuperior quadrants.
3. With acetabular anteversion increased to 30°, SF values dropped to 2–3 in the anterosuperior and posterosuperior quadrants.
4. With acetabular anteversion reduced to 0°, SF values fell below 5, and locally to 2–3 in the posterosuperior quadrant.
5. With an increased femoral head offset of 1 cm, SF values decreased to 3–4 in the anterosuperior and anteroinferior quadrants of the acetabulum.

## 4. Discussion

In most of the cases dislocation of the prosthesis is attributed to technical errors during implant placement, particularly inaccuracies in the orientation of the acetabular component. Consequently, preventive measures against this complication focus both on ensuring proper implant positioning and selecting the most appropriate implant type for a given clinical scenario [10]. Increasingly, constrained liners and dual mobility cups are being utilized, as they enhance joint congruency and thereby reduce the risk of dislocation [11]. Dual mobility acetabular components combine a small prosthetic head that articulates within a mobile polyethylene liner, which itself is free to move within the metal acetabular shell. This design not only minimizes the risk of dislocation but also increases the range of motion of the prosthetic joint, which is particularly advantageous in elderly patients and those at high risk of joint instability [12].

The efficacy and utility of such solutions are most frequently assessed retrospectively by analyzing dislocation rates. Mathematical modeling offers an alternative approach, enabling prediction of implant behavior through analysis of the forces acting upon its components [13,14]. The use of dedicated engineering software allows for the creation of a mathematical model of the hip joint, in which various prosthesis configurations can be analyzed to assess their associated benefits and risks [15,16].

In the present study a biomechanical mathematical model was developed based on the finite element method. It incorporated anatomical features of the hip joint as well as muscle activity. The objective of the model was to assess the Safety Factor and to analyze how different prosthesis configuration, including offset, anteversion, inclination, and femoral stem depth, influence joint’s stability. Obtained results confirm that proper alignment of the prosthetic components, both the femoral stem and the acetabular cup, significantly reduce the risk of dislocation after total hip arthroplasty.

The analysis demonstrated a substantial impact of implant’s positioning on its stability, expressed by value of the Safety Factor. Only the configuration deemed optimal, in which the acetabular cup is placed at 45° inclination and 15° anteversion, with the prosthetic axis of rotation aligned with the contralateral hip joint, offset ensuring symmetry in mechanical axis displacement relative to the body’s midline, and stem positioning ensuring equal leg length resulted in biomechanical equilibrium of the acting forces. This was reflected in high SF values ranging between 9 and 12 indicating low risk of dislocation, loosening, or component failure. Other configurations with deviations in inclination, anteversion, stem depth, or offset caused a significant reduction in SF values to the range of 2–5, being most strongly expressed in the antero-superior and postero-superior quadrants of the acetabulum.

**Figure 4:**
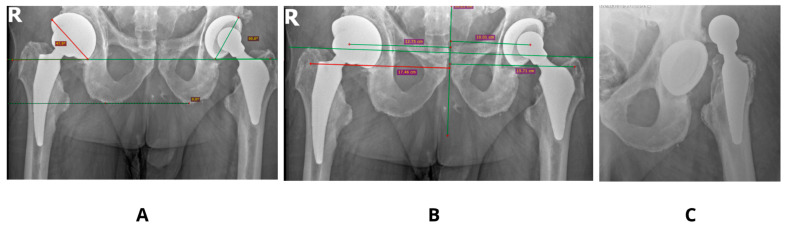
Clinical case showing an acetabulum with increased anteversion and inclination angle 60°—(**A**,**B**). Dislocation of the same prosthesis—(**C**).

Literature examples emphasizes that precise acetabular positioning within the so-called functional safe zone is essential for minimizing biomechanical complications. According to Teja et al. [17] even minor deviations outside the 30–50° inclination and 5–25° anteversion range can impair prosthesis biomechanics and compromise functional stability. Observed in our studies decrease in SF to values as low as 2–3 in models with increased anteversion (30°) and shallow stem insertion highlights the sensitivity of these parameters and aligns with clinical findings, finding its confirmation in our clinical observations [Figure 4].

Optimizing implant positioning is crucial for ensuring mechanical stability, and in this study, a configuration involving 45° of acetabular inclination, 15° of anteversion, standard femoral offset, and appropriate stem insertion depth was associated with favorable Safety Factor (SF) values. Nevertheless, such outcomes must be interpreted within the context of individual biomechanical variability.

**Figure 5:**
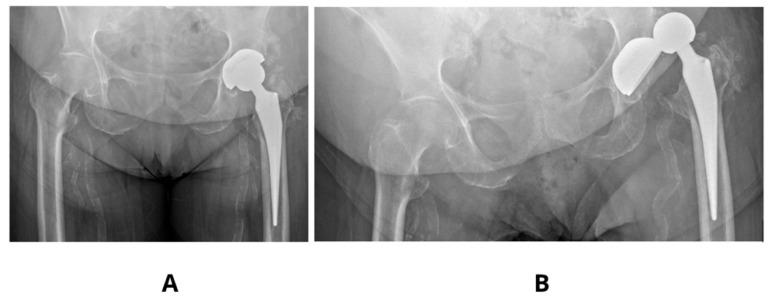
An example of an implant inserted with 0° anteversion of the acetabulum—(**A**), and its dislocation—(**B**).

Patient-specific factors, including pelvic tilt and femoral morphology (e.g., neck-shaft angle, femoral version, and offset), substantially influence prosthetic component orientation and joint stability. Prior research by Yang et al. [18] demonstrated that minor alterations in pelvic tilt (5–10°) can significantly impact functional anteversion, increasing the risk of prosthetic impingement or dislocation [Figure 5].

Moreover, postoperative changes in pelvic alignment—resulting from neuromuscular adaptation or compensatory postural adjustments—can secondarily increase acetabular inclination and anteversion, thereby lowering local SF values. This is consistent with our findings, in which increased anteversion was associated with SF values as low as 2–3.

These findings reinforce the need for individualized surgical planning that incorporates not only anatomical variation but also dynamic postural factors, to enhance implant longevity and reduce the incidence of mechanical complications.

A review of literature on the biomechanical impact of increased femoral offset after arthroplasty reveals a notable correlation between experimental and clinical outcomes. While increasing femoral head offset improves joint stability, it may also elevate rotational loads, reducing SF at the prosthesis-liner interface, particularly in the anterosuperior and anteroinferior quadrants of the acetabulum.

Shimizu et al. [19] found that increasing femoral neck offset improves joint stability, but excessive offset leads to higher abductor muscle moments, thereby increasing load on the prosthetic joint. Similarly, Bahl et al. [20] showed that a longer abductor lever arm generates greater torque, resulting in higher contact forces and potentially reduced SF at the head–liner interface.

Avhad et al. [21] demonstrated that although high offset can improve joint function and reduce the risk of dislocation, an excessive offset increases the moment of flexion forces and imposes disproportionate loading on the acetabular component. These changes may degrade the component’s congruency and increase the risk of loosening, which is expressed by SF values dropping below 4 an, pointing out the increased risk of dislocation.

The use of assistive technologies, such as computer navigation and robotics, significantly increases the precision of positioning endoprosthesis components. Zhang et al. [22] reported that navigation systems reduce the incidence of cup mispositioning by up to 50%, which translates into higher and more uniformly distributed postoperative SF values. This highlights the clinical necessity of using advanced technologies to maintain optimal implant’s safety margins.

The lowest observed SF values (2–3), which are associated with a high risk of implant dislocation, are consistent with findings by Maratt and D’Lima [23], and Widmer [24]. Their studies confirm that both increased and decreased anteversion adversely affect hip prosthesis stability and substantially increase the risk of dislocation.

A limitation of this study was the exclusive analysis of single-leg stance with body weight loading during upright posture. We have also decided not to analyze observed in our studies lowering of SF in other than acetabular insert—prosthetic head parts of the implant, focusing on the mechanism of implant’s dislocation. Nevertheless, as could be observed in Figure 3 their lowering up to 2–3 threatening implant’s loosing (at the border between acetabular cup and pelvic bone, as well as around the medical aspect of the femoral stem) or periprosthetic bone fracture (at the apex of the stem).

The SF in this study was assessed under static loading conditions simulating single-leg stance. Despite the simplified loading scenario, low SF values (e.g., <3) may indicate areas of mechanical vulnerability with limited capacity to accommodate increased stress. In clinical practice, dislocations frequently occur during dynamic activities or accidental trauma, where joint loads can rise sharply within milliseconds. Under such circumstances, implant configurations operating close to their structural limits are at markedly increased risk of micromotion, interface failure, or dislocation. Consequently, SF can be interpreted as a surrogate marker of biomechanical resilience, reflecting the implant’s ability to withstand both physiological and non-physiological loading conditions.

Clinical observations suggest that dislocations in most cases take place on activities that significantly differ from this model, such as falling from the bed, slipping, descending the stairs, or due to other trauma-related events, where limb’s positioning and muscle’s tensions are difficult to predict [25]. Particularly vulnerable are patients with impaired cognition (e.g., due to dementia) or those under the influence of alcohol or psychoactive substances. In this particular group of patients, the postoperative risk of dislocation may be up to four times higher than that in the general population [26].

While an optimal implant positioning significantly reduces risk of dislocation, postoperative factors—including patient compliance, physical therapy protocols, and cognitive or functional impairments—are equally critical. Clinical observations and literature data suggest that in most cases dislocations occur due to unpredictable activities. Therefore, patient’s education, an appropriate rehabilitation, and tailored movement restrictions must be an integral component of postoperative care supporting the mechanical stability of the implant. Future biomechanical studies could investigate the effects of typical at-risk patient movements during rehabilitation, such as flexion combined with internal rotation, which are known to be responsible for posterior dislocation.

In summary, our results confirm the critical importance of proper prosthesis component positioning for maintaining high Safety Factor values. Furthermore, the identification of mechanically vulnerable regions (e.g., prosthetic head–liner interface, anterosuperior acetabular quadrant, stem tip) is essential for optimizing treatment strategies and postoperative monitoring. This underscores the need for precise preoperative planning and the intraoperative use of support technologies in clinical practice.

One of the most important limitations of this study is the use of a single anatomical model derived from a CT scan of an individual with normal hip morphology. While this enabled a standardized analysis of implant positioning, it does not capture the anatomical variability seen in clinical populations. In particular, patients with osteoporosis or hip dysplasia may exhibit markedly different bone quality and joint geometry, which can significantly affect load transmission, implant fixation, and the resulting biomechanical safety margins. Future studies should incorporate population-specific anatomical datasets or parametric modeling approaches to account for such variability and improve clinical applicability.

## 5. Conclusions

- Proper positioning of the hip endoprosthesis promotes an even distribution of the Safety Factor within the acetabular component.
- The lowest Safety Factor values (2–3), associated with an increased risk of implant dislocation, are observed in cases of insufficient femoral stem insertion (increased risk of anterior dislocation), excessive acetabular anteversion (increased risk of anterior dislocation), and reduced acetabular anteversion (increased risk of posterior dislocation).
- Deviations from standard implant positioning, both in terms of femoral stem depth and acetabular cup orientation (inclination, anteversion), lead to a decrease in the Safety Factor.
- Alterations in femoral head offset reduce the Safety Factor specifically within the anterior quadrants of the acetabulum.
- Use of assistive technologies such as robotic and navigation systems significantly improves implant positioning accuracy, which translates into higher Safety Factor values and lower dislocation risk.
- In high-risk patients, computer-assisted THA may provide biomechanical advantages by minimizing malposition-related overloads and enhancing long-term implant stability.

## Data Availability

The data presented in this study are available on request from the corresponding author.

## Abbreviations

The following abbreviations are used in this manuscript:

THA	Total hip arthroplasty
SF	Safety Factor
FEM	Finite element method
N	Newtons
CT	Computer tomography
